# Bis{μ-2-(1*H*-indol-3-yl)-*N*′-[1-(5-methyl-2-oxidophen­yl)ethyl­idene]­aceto­hydraz­idato}bis­[aqua­zinc(II)] dimethyl sulfoxide tetra­solvate

**DOI:** 10.1107/S1600536808022034

**Published:** 2008-07-19

**Authors:** Kadir Zuraini, Hapipah M. Ali, Subramaniam Puvaneswary, Ward T. Robinson, Seik Weng Ng

**Affiliations:** a Department of Chemistry, University of Malaya, 50603 Kuala Lumpur, Malaysia

## Abstract

The dinuclear title compound, [Zn_2_(C_19_H_17_N_3_O_2_)_2_(H_2_O)_2_]·4C_2_H_6_OS, lies about a center of inversion. The deprotonated monoanion *O*,*N*,*O-*chelates the Zn atom; the hydr­oxy O atom also engages in bonding to the symmetry-related Zn atom so that one N and three O atoms form a square around the metal. The coordination geometry is square-pyramidal, with the apical site occupied by a water mol­ecule. Hydrogen bonds, with the water mol­ecule serving as donor atom, lead to the formation of a linear chain motif. There is an N—H⋯O hydrogen bond between the complex molecule and solvent O atom.

## Related literature

For the structure of a similar Schiff base ligand, see: Ali *et al.* (2008 ▶).
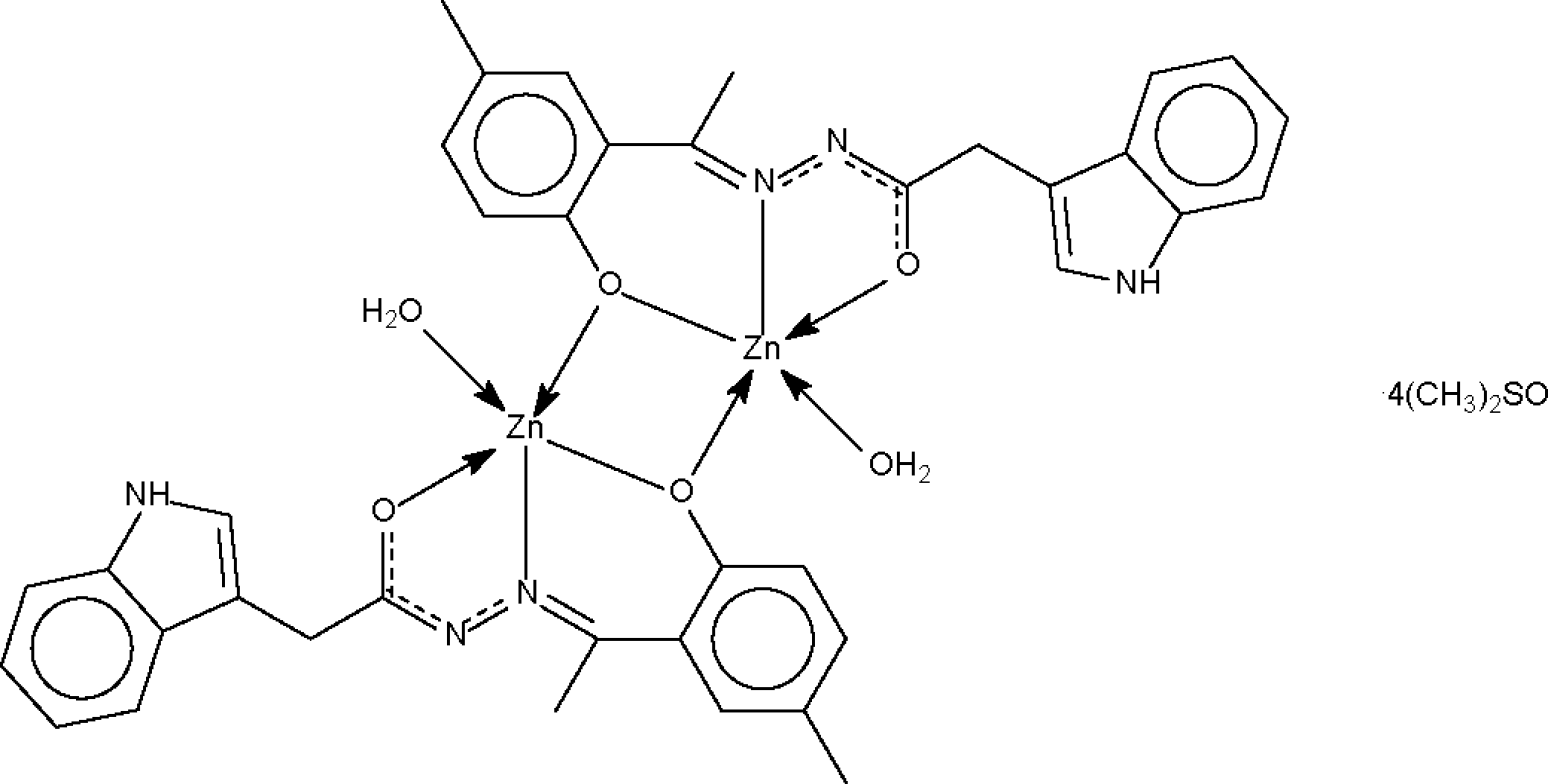


## Experimental

### 

#### Crystal data

[Zn_2_(C_19_H_17_N_3_O_2_)_2_(H_2_O)_2_]·4C_2_H_6_OS
*M* *_r_* = 1118.00
Triclinic, 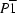

*a* = 8.5271 (2) Å
*b* = 8.8849 (3) Å
*c* = 16.8279 (5) Å
α = 85.519 (2)°
β = 84.920 (2)°
γ = 84.251 (2)°
*V* = 1260.44 (6) Å^3^
*Z* = 1
Mo *K*α radiation
μ = 1.18 mm^−1^
*T* = 100 (2) K
0.19 × 0.03 × 0.03 mm

#### Data collection

Bruker SMART APEX diffractometer
Absorption correction: multi-scan (*SADABS*; Sheldrick, 1996 ▶) *T*_min_ = 0.807, *T*_max_ = 0.966
13526 measured reflections
5739 independent reflections
3700 reflections with *I* > 2σ(*I*)
*R*_int_ = 0.076

#### Refinement

*R*[*F* ^2^ > 2σ(*F* ^2^)] = 0.056
*wR*(*F* ^2^) = 0.157
*S* = 0.99
5739 reflections
313 parameters
H-atom parameters constrained
Δρ_max_ = 1.18 e Å^−3^
Δρ_min_ = −1.24 e Å^−3^

### 

Data collection: *APEX2* (Bruker, 2007 ▶); cell refinement: *SAINT* (Bruker, 2007 ▶); data reduction: *SAINT*; program(s) used to solve structure: *SHELXS97* (Sheldrick, 2008 ▶); program(s) used to refine structure: *SHELXL97* (Sheldrick, 2008 ▶); molecular graphics: *X-SEED* (Barbour, 2001 ▶); software used to prepare material for publication: *publCIF* (Westrip, 2008 ▶).

## Supplementary Material

Crystal structure: contains datablocks global, I. DOI: 10.1107/S1600536808022034/si2100sup1.cif

Structure factors: contains datablocks I. DOI: 10.1107/S1600536808022034/si2100Isup2.hkl

Additional supplementary materials:  crystallographic information; 3D view; checkCIF report

## Figures and Tables

**Table 1:** Hydrogen-bond geometry (Å, °)

*D*—H⋯*A*	*D*—H	H⋯*A*	*D*⋯*A*	*D*—H⋯*A*
O1*W*—H1*W*1⋯O3	0.84	1.80	2.608 (4)	161
O1*W*—H1*W*2⋯N2^i^	0.84	1.87	2.703 (4)	173
N3—H3N⋯O4	0.88	1.95	2.822 (5)	172

Symmetry code: (i) 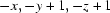.

**Fig. 1.**
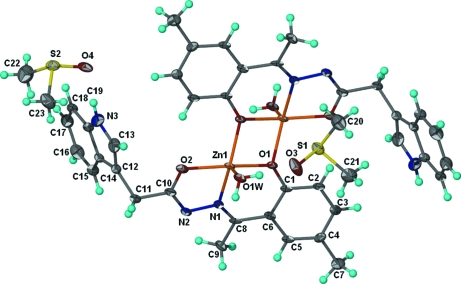
Thermal ellipsoid plot (Barbour, 2001) plot of the dinuclear compound at the 70% probability level. Hydrogen atoms are drawn as spheres of arbitrary radius. The compound lies about a center of inversion, and unlabeled atoms are related to their labeled equivalents by 1 – x, 1 – y, 1 – z.

## supplementary crystallographic information

### Comment

The present study continues with a study on 1-(2-hydroxy-5-methylphenyl)ethanone [(1*H*-3-indolyl)acetyl]hydrazone (Ali *et al.*, 2008). The present study reports the zinc derivative of a similar ligand (Scheme I, Fig. 1). The dinuclear compound lies about a center-of-inversion. The deprotonated monoanion *O*,*N*,*O* chelates to the Zn atom; the hydroxy oxygen atom also engages in bonding to the symmetry-related Zn atom so that one nitrogen and oxygen atoms comprise a square around the metal. The geometry is a square pyramid, with the apical site occupied by a water molecule. Hydrogen bonds, with the water molecule serving as donor atom, leads to the formation of a linear chain motif.

### Experimental

Indole-3- acetic acid hydrazide (0.55 g, 4 mmol) and 5-methyl-2-hydroxyacetophenone (0.52 g, 4 mmol) were heated in ethanol for 2 h. The solvent was removed to give the Schiff base. The ligand (0.55 g, 4 mmol) and zinc acetate (0.26 g, 2 mmol) were dissolved in basified ethanol and heated for 5 h. The yellow solid was that was obtained was recrystallized from DMSO.

### Refinement

Carbon and nitrogen-bound H-atoms were placed in calculated positions (C—H 0.95 to 0.98 Å, N–H 0.88 Å) and were included in the refinement in the riding model approximation, with *U*_iso_(H) set to 1.2 to 1.5*U*_eq_(C). The water H-atoms were placed in chemically sensible positions on the basis of hydrogen bonding, but were not refined.

The final difference Fourier map had a large peak/deep hole near Zn1.

### Crystal data

**Table:**

[Zn_2_(C_19_H_17_N_3_O_2_)_2_(H_2_O)_2_]·4C_2_H_6_OS	*Z* = 1
*M**_r_* = 1118.00	*F*_000_ = 584
Triclinic, *P*1	*D*_x_ = 1.473 Mg m^−^^3^
Hall symbol: -P 1	Mo *K*α radiation λ = 0.71073 Å
*a* = 8.5271 (2) Å	Cell parameters from 2055 reflections
*b* = 8.8849 (3) Å	θ = 2.4–21.0º
*c* = 16.8279 (5) Å	µ = 1.18 mm^−^^1^
α = 85.519 (2)º	*T* = 100 (2) K
β = 84.920 (2)º	Block, yellow
γ = 84.251 (2)º	0.19 × 0.03 × 0.03 mm
*V* = 1260.44 (6) Å^3^	

### Data collection

**Table:**

Bruker SMART APEX diffractometer	5739 independent reflections
Radiation source: fine-focus sealed tube	3700 reflections with *I* > 2σ(*I*)
Monochromator: graphite	*R*_int_ = 0.077
*T* = 100(2) K	θ_max_ = 27.5º
ω scans	θ_min_ = 1.2º
Absorption correction: Multi-scan(SADABS; Sheldrick, 1996)	*h* = −11→11
*T*_min_ = 0.807, *T*_max_ = 0.966	*k* = −11→9
13526 measured reflections	*l* = −21→21

### Refinement

**Table:**

Refinement on *F*^2^	Secondary atom site location: difference Fourier map
Least-squares matrix: full	Hydrogen site location: inferred from neighbouring sites
*R*[*F*^2^ > 2σ(*F*^2^)] = 0.056	H-atom parameters constrained
*wR*(*F*^2^) = 0.157	*w* = 1/[σ^2^(*F*_o_^2^) + (0.0772*P*)^2^] where *P* = (*F*_o_^2^ + 2*F*_c_^2^)/3
*S* = 0.99	(Δ/σ)_max_ = 0.001
5739 reflections	Δρ_max_ = 1.18 e Å^−^^3^
313 parameters	Δρ_min_ = −1.24 e Å^−^^3^
Primary atom site location: structure-invariant direct methods	Extinction correction: none

### Fractional atomic coordinates and isotropic or equivalent isotropic displacement parameters (Å^2^)

**Table:**

	*x*	*y*	*z*	*U*_iso_*/*U*_eq_	
Zn1	0.32563 (6)	0.51110 (6)	0.47468 (3)	0.01466 (16)	
S2	0.28628 (14)	0.71165 (15)	−0.05107 (7)	0.0237 (3)	
S1	0.51759 (13)	0.81274 (15)	0.61958 (7)	0.0223 (3)	
O1	0.4553 (3)	0.4370 (4)	0.56890 (17)	0.0159 (7)	
O2	0.2019 (3)	0.5011 (4)	0.37781 (18)	0.0213 (7)	
O3	0.3597 (4)	0.8802 (4)	0.5929 (2)	0.0338 (9)	
O4	0.3710 (4)	0.6688 (4)	0.02322 (19)	0.0237 (8)	
O1W	0.2288 (3)	0.7174 (4)	0.49948 (18)	0.0198 (7)	
H1W1	0.2774	0.7502	0.5348	0.030*	
H1W2	0.1334	0.7129	0.5162	0.030*	
N1	0.1821 (4)	0.3402 (4)	0.5139 (2)	0.0135 (8)	
N2	0.0796 (4)	0.3130 (4)	0.4563 (2)	0.0147 (8)	
N3	0.2308 (5)	0.4574 (5)	0.1345 (2)	0.0264 (10)	
H3N	0.2808	0.5159	0.0981	0.032*	
C1	0.4099 (5)	0.3577 (5)	0.6363 (2)	0.0144 (9)	
C2	0.4983 (5)	0.3584 (5)	0.7028 (2)	0.0179 (10)	
H2	0.5895	0.4127	0.6978	0.021*	
C3	0.4569 (5)	0.2829 (5)	0.7751 (3)	0.0180 (10)	
H3	0.5204	0.2848	0.8185	0.022*	
C4	0.3218 (5)	0.2035 (5)	0.7849 (2)	0.0173 (9)	
C5	0.2384 (5)	0.1992 (5)	0.7189 (2)	0.0158 (9)	
H5	0.1486	0.1428	0.7247	0.019*	
C6	0.2765 (5)	0.2724 (5)	0.6434 (2)	0.0148 (9)	
C7	0.2737 (6)	0.1222 (6)	0.8645 (3)	0.0255 (11)	
H7A	0.1851	0.0629	0.8584	0.038*	
H7B	0.2415	0.1969	0.9041	0.038*	
H7C	0.3635	0.0543	0.8824	0.038*	
C8	0.1777 (5)	0.2507 (5)	0.5786 (2)	0.0139 (9)	
C9	0.0720 (5)	0.1235 (5)	0.5894 (3)	0.0201 (10)	
H9A	0.0518	0.0925	0.5369	0.030*	
H9B	−0.0285	0.1580	0.6182	0.030*	
H9C	0.1237	0.0371	0.6200	0.030*	
C10	0.1006 (5)	0.4035 (5)	0.3904 (3)	0.0166 (9)	
C11	−0.0086 (5)	0.3896 (5)	0.3254 (2)	0.0170 (10)	
H11A	−0.0626	0.2958	0.3372	0.020*	
H11B	−0.0906	0.4768	0.3252	0.020*	
C12	0.0803 (5)	0.3853 (5)	0.2444 (2)	0.0166 (9)	
C13	0.1430 (6)	0.5047 (6)	0.2012 (3)	0.0244 (11)	
H13	0.1275	0.6067	0.2156	0.029*	
C14	0.1322 (5)	0.2547 (5)	0.2012 (2)	0.0164 (9)	
C15	0.1120 (5)	0.0994 (6)	0.2144 (3)	0.0207 (10)	
H15	0.0459	0.0636	0.2588	0.025*	
C16	0.1893 (6)	0.0001 (6)	0.1621 (3)	0.0265 (11)	
H16	0.1765	−0.1051	0.1710	0.032*	
C17	0.2869 (5)	0.0515 (6)	0.0958 (3)	0.0261 (11)	
H17	0.3397	−0.0200	0.0610	0.031*	
C18	0.3076 (5)	0.2019 (6)	0.0802 (3)	0.0230 (11)	
H18	0.3729	0.2365	0.0351	0.028*	
C19	0.2282 (5)	0.3032 (6)	0.1335 (3)	0.0193 (10)	
C20	0.6333 (6)	0.9686 (6)	0.6179 (3)	0.0312 (12)	
H20A	0.6492	1.0129	0.5630	0.047*	
H20B	0.7361	0.9336	0.6380	0.047*	
H20C	0.5784	1.0453	0.6519	0.047*	
C21	0.4833 (7)	0.7774 (8)	0.7244 (3)	0.0400 (15)	
H21A	0.4050	0.7035	0.7359	0.060*	
H21B	0.4436	0.8722	0.7485	0.060*	
H21C	0.5825	0.7370	0.7468	0.060*	
C22	0.2380 (7)	0.5408 (7)	−0.0869 (3)	0.0395 (14)	
H22A	0.3354	0.4786	−0.1030	0.059*	
H22B	0.1751	0.5646	−0.1330	0.059*	
H22C	0.1768	0.4848	−0.0444	0.059*	
C23	0.0906 (6)	0.7843 (7)	−0.0178 (3)	0.0329 (13)	
H23A	0.0953	0.8749	0.0113	0.049*	
H23B	0.0403	0.7071	0.0177	0.049*	
H23C	0.0287	0.8109	−0.0641	0.049*	

### Atomic displacement parameters (Å^2^)

**Table:**

	*U*^11^	*U*^22^	*U*^33^	*U*^12^	*U*^13^	*U*^23^
Zn1	0.0079 (2)	0.0202 (3)	0.0158 (3)	−0.0058 (2)	0.00131 (18)	0.0025 (2)
S2	0.0200 (6)	0.0270 (7)	0.0230 (6)	−0.0063 (5)	0.0020 (5)	0.0058 (5)
S1	0.0161 (5)	0.0233 (7)	0.0278 (6)	0.0003 (5)	−0.0040 (5)	−0.0040 (5)
O1	0.0111 (14)	0.0222 (19)	0.0141 (14)	−0.0070 (13)	0.0009 (12)	0.0050 (13)
O2	0.0153 (15)	0.030 (2)	0.0195 (16)	−0.0110 (14)	0.0012 (13)	0.0041 (14)
O3	0.0222 (18)	0.035 (2)	0.047 (2)	0.0040 (16)	−0.0174 (17)	−0.0109 (18)
O4	0.0163 (16)	0.025 (2)	0.0291 (18)	−0.0088 (14)	0.0001 (14)	0.0103 (15)
O1W	0.0107 (14)	0.0250 (19)	0.0238 (16)	−0.0057 (13)	−0.0002 (13)	0.0012 (14)
N1	0.0096 (16)	0.017 (2)	0.0142 (17)	−0.0018 (15)	0.0008 (14)	−0.0016 (15)
N2	0.0096 (16)	0.017 (2)	0.0178 (18)	−0.0033 (15)	0.0011 (14)	−0.0021 (15)
N3	0.034 (2)	0.028 (3)	0.018 (2)	−0.014 (2)	−0.0004 (18)	0.0056 (18)
C1	0.0089 (19)	0.016 (2)	0.017 (2)	−0.0029 (17)	0.0038 (16)	0.0011 (17)
C2	0.011 (2)	0.024 (3)	0.019 (2)	−0.0063 (19)	0.0049 (17)	−0.0035 (19)
C3	0.016 (2)	0.021 (3)	0.017 (2)	−0.0057 (19)	−0.0003 (18)	0.0014 (18)
C4	0.019 (2)	0.019 (3)	0.013 (2)	−0.0018 (19)	0.0021 (17)	0.0032 (18)
C5	0.010 (2)	0.017 (3)	0.019 (2)	−0.0036 (18)	0.0036 (17)	0.0028 (18)
C6	0.0098 (19)	0.015 (2)	0.019 (2)	−0.0030 (17)	0.0002 (17)	0.0018 (18)
C7	0.026 (3)	0.028 (3)	0.022 (2)	−0.010 (2)	0.000 (2)	0.006 (2)
C8	0.0078 (19)	0.013 (2)	0.019 (2)	−0.0016 (17)	0.0057 (16)	0.0034 (18)
C9	0.014 (2)	0.024 (3)	0.023 (2)	−0.009 (2)	−0.0042 (18)	0.003 (2)
C10	0.0089 (19)	0.021 (3)	0.020 (2)	−0.0030 (18)	0.0020 (17)	−0.0019 (19)
C11	0.0093 (19)	0.024 (3)	0.018 (2)	−0.0017 (19)	0.0008 (17)	−0.0018 (19)
C12	0.014 (2)	0.022 (3)	0.014 (2)	−0.0057 (19)	−0.0026 (17)	0.0037 (18)
C13	0.031 (3)	0.022 (3)	0.021 (2)	−0.007 (2)	−0.007 (2)	0.003 (2)
C14	0.011 (2)	0.024 (3)	0.015 (2)	−0.0040 (19)	−0.0031 (17)	0.0025 (18)
C15	0.018 (2)	0.024 (3)	0.020 (2)	−0.006 (2)	−0.0002 (18)	−0.0007 (19)
C16	0.027 (3)	0.018 (3)	0.035 (3)	−0.004 (2)	−0.007 (2)	0.000 (2)
C17	0.021 (2)	0.030 (3)	0.028 (3)	−0.001 (2)	−0.002 (2)	−0.010 (2)
C18	0.012 (2)	0.039 (3)	0.019 (2)	−0.006 (2)	0.0002 (18)	−0.005 (2)
C19	0.015 (2)	0.028 (3)	0.016 (2)	−0.008 (2)	−0.0007 (17)	0.0033 (19)
C20	0.029 (3)	0.027 (3)	0.039 (3)	−0.002 (2)	−0.009 (2)	0.000 (2)
C21	0.031 (3)	0.058 (4)	0.031 (3)	−0.010 (3)	−0.001 (2)	0.005 (3)
C22	0.048 (4)	0.035 (4)	0.036 (3)	−0.004 (3)	−0.003 (3)	−0.009 (3)
C23	0.020 (3)	0.036 (3)	0.043 (3)	0.002 (2)	−0.009 (2)	−0.005 (3)

### Geometric parameters (Å, °)

**Table:**

Zn1—O1	2.043 (3)	C7—H7B	0.9800
Zn1—O1^i^	2.029 (3)	C7—H7C	0.9800
Zn1—O2	2.031 (3)	C8—C9	1.506 (6)
Zn1—O1w	1.993 (3)	C9—H9A	0.9800
Zn1—N1	2.077 (4)	C9—H9B	0.9800
Zn1—Zn1^i^	3.1485 (9)	C9—H9C	0.9800
S2—O4	1.503 (3)	C10—C11	1.518 (5)
S2—C22	1.775 (6)	C11—C12	1.502 (6)
S2—C23	1.786 (5)	C11—H11A	0.9900
S1—O3	1.509 (3)	C11—H11B	0.9900
S1—C21	1.771 (5)	C12—C13	1.365 (6)
S1—C20	1.776 (5)	C12—C14	1.428 (6)
O1—C1	1.335 (5)	C13—H13	0.9500
O1—Zn1^i^	2.029 (3)	C14—C15	1.406 (6)
O2—C10	1.277 (5)	C14—C19	1.408 (6)
O1W—H1W1	0.8400	C15—C16	1.374 (7)
O1W—H1W2	0.8400	C15—H15	0.9500
N1—C8	1.297 (5)	C16—C17	1.408 (7)
N1—N2	1.410 (4)	C16—H16	0.9500
N2—C10	1.328 (6)	C17—C18	1.368 (7)
N3—C13	1.363 (6)	C17—H17	0.9500
N3—C19	1.374 (6)	C18—C19	1.404 (7)
N3—H3N	0.8800	C18—H18	0.9500
C1—C2	1.404 (6)	C20—H20A	0.9800
C1—C6	1.420 (6)	C20—H20B	0.9800
C2—C3	1.377 (6)	C20—H20C	0.9800
C2—H2	0.9500	C21—H21A	0.9800
C3—C4	1.401 (6)	C21—H21B	0.9800
C3—H3	0.9500	C21—H21C	0.9800
C4—C5	1.375 (6)	C22—H22A	0.9800
C4—C7	1.515 (6)	C22—H22B	0.9800
C5—C6	1.408 (6)	C22—H22C	0.9800
C5—H5	0.9500	C23—H23A	0.9800
C6—C8	1.472 (5)	C23—H23B	0.9800
C7—H7A	0.9800	C23—H23C	0.9800
			
O1—Zn1—O1^i^	78.7 (1)	C8—C9—H9B	109.5
O1—Zn1—O2	158.8 (1)	H9A—C9—H9B	109.5
O1—Zn1—O1w	105.3 (1)	C8—C9—H9C	109.5
O1—Zn1—N1	86.3 (1)	H9A—C9—H9C	109.5
O1^i^—Zn1—O2	106.0 (1)	H9B—C9—H9C	109.5
O1^i^—Zn1—O1w	99.5 (1)	O2—C10—N2	126.0 (4)
O1^i^—Zn1—N1	146.5 (1)	O2—C10—C11	118.0 (4)
O2—Zn1—O1w	94.5 (1)	N2—C10—C11	116.0 (4)
O2—Zn1—N1	78.7 (1)	C12—C11—C10	111.6 (3)
O1W—Zn1—N1	113.4 (1)	C12—C11—H11A	109.3
O1W—Zn1—Zn1^i^	106.07 (8)	C10—C11—H11A	109.3
O1^i^—Zn1—Zn1^i^	39.51 (8)	C12—C11—H11B	109.3
O2—Zn1—Zn1^i^	141.51 (9)	C10—C11—H11B	109.3
O1—Zn1—Zn1^i^	39.19 (8)	H11A—C11—H11B	108.0
N1—Zn1—Zn1^i^	119.67 (10)	C13—C12—C14	106.0 (4)
O4—S2—C22	107.0 (2)	C13—C12—C11	126.2 (4)
O4—S2—C23	106.1 (2)	C14—C12—C11	127.4 (4)
C22—S2—C23	97.8 (3)	N3—C13—C12	110.7 (5)
O3—S1—C21	104.4 (2)	N3—C13—H13	124.7
O3—S1—C20	105.1 (2)	C12—C13—H13	124.7
C21—S1—C20	99.1 (3)	C15—C14—C19	118.5 (4)
C1—O1—Zn1^i^	129.8 (2)	C15—C14—C12	134.2 (4)
C1—O1—Zn1	128.3 (2)	C19—C14—C12	107.2 (4)
Zn1^i^—O1—Zn1	101.30 (12)	C16—C15—C14	119.1 (4)
C10—O2—Zn1	111.6 (3)	C16—C15—H15	120.4
Zn1—O1W—H1W1	109.5	C14—C15—H15	120.4
Zn1—O1W—H1W2	109.5	C15—C16—C17	121.1 (5)
H1W1—O1W—H1W2	109.5	C15—C16—H16	119.4
C8—N1—N2	116.0 (4)	C17—C16—H16	119.4
C8—N1—Zn1	131.5 (3)	C18—C17—C16	121.5 (5)
N2—N1—Zn1	112.4 (3)	C18—C17—H17	119.2
C10—N2—N1	111.2 (3)	C16—C17—H17	119.2
C13—N3—C19	108.6 (4)	C17—C18—C19	117.2 (4)
C13—N3—H3N	125.7	C17—C18—H18	121.4
C19—N3—H3N	125.7	C19—C18—H18	121.4
O1—C1—C2	118.6 (4)	N3—C19—C18	130.0 (4)
O1—C1—C6	122.8 (4)	N3—C19—C14	107.5 (4)
C2—C1—C6	118.6 (4)	C18—C19—C14	122.5 (5)
C3—C2—C1	122.1 (4)	S1—C20—H20A	109.5
C3—C2—H2	118.9	S1—C20—H20B	109.5
C1—C2—H2	118.9	H20A—C20—H20B	109.5
C2—C3—C4	120.4 (4)	S1—C20—H20C	109.5
C2—C3—H3	119.8	H20A—C20—H20C	109.5
C4—C3—H3	119.8	H20B—C20—H20C	109.5
C5—C4—C3	117.2 (4)	S1—C21—H21A	109.5
C5—C4—C7	121.9 (4)	S1—C21—H21B	109.5
C3—C4—C7	120.9 (4)	H21A—C21—H21B	109.5
C4—C5—C6	124.7 (4)	S1—C21—H21C	109.5
C4—C5—H5	117.7	H21A—C21—H21C	109.5
C6—C5—H5	117.7	H21B—C21—H21C	109.5
C5—C6—C1	116.8 (4)	S2—C22—H22A	109.5
C5—C6—C8	117.3 (4)	S2—C22—H22B	109.5
C1—C6—C8	125.8 (4)	H22A—C22—H22B	109.5
C4—C7—H7A	109.5	S2—C22—H22C	109.5
C4—C7—H7B	109.5	H22A—C22—H22C	109.5
H7A—C7—H7B	109.5	H22B—C22—H22C	109.5
C4—C7—H7C	109.5	S2—C23—H23A	109.5
H7A—C7—H7C	109.5	S2—C23—H23B	109.5
H7B—C7—H7C	109.5	H23A—C23—H23B	109.5
N1—C8—C6	120.3 (4)	S2—C23—H23C	109.5
N1—C8—C9	121.1 (4)	H23A—C23—H23C	109.5
C6—C8—C9	118.6 (4)	H23B—C23—H23C	109.5
C8—C9—H9A	109.5		
			
O1W—Zn1—O1—C1	91.0 (4)	C2—C1—C6—C5	2.7 (6)
O1^i^—Zn1—O1—C1	−172.2 (4)	O1—C1—C6—C8	4.2 (7)
O2—Zn1—O1—C1	−66.9 (5)	C2—C1—C6—C8	−175.6 (4)
N1—Zn1—O1—C1	−22.3 (4)	N2—N1—C8—C6	−178.2 (4)
Zn1^i^—Zn1—O1—C1	−172.2 (4)	Zn1—N1—C8—C6	6.6 (6)
O1W—Zn1—O1—Zn1^i^	−96.87 (14)	N2—N1—C8—C9	1.6 (6)
O1^i^—Zn1—O1—Zn1^i^	0.0	Zn1—N1—C8—C9	−173.6 (3)
O2—Zn1—O1—Zn1^i^	105.2 (3)	C5—C6—C8—N1	164.2 (4)
N1—Zn1—O1—Zn1^i^	149.86 (15)	C1—C6—C8—N1	−17.5 (7)
O1W—Zn1—O2—C10	−111.0 (3)	C5—C6—C8—C9	−15.6 (6)
O1^i^—Zn1—O2—C10	147.8 (3)	C1—C6—C8—C9	162.7 (4)
O1—Zn1—O2—C10	47.6 (5)	Zn1—O2—C10—N2	−2.8 (6)
N1—Zn1—O2—C10	2.0 (3)	Zn1—O2—C10—C11	176.2 (3)
Zn1^i^—Zn1—O2—C10	126.0 (3)	N1—N2—C10—O2	1.7 (6)
O1W—Zn1—N1—C8	−95.9 (4)	N1—N2—C10—C11	−177.3 (3)
O1^i^—Zn1—N1—C8	72.1 (5)	O2—C10—C11—C12	45.9 (6)
O2—Zn1—N1—C8	174.1 (4)	N2—C10—C11—C12	−135.0 (4)
O1—Zn1—N1—C8	9.1 (4)	C10—C11—C12—C13	−72.6 (6)
Zn1^i^—Zn1—N1—C8	30.5 (4)	C10—C11—C12—C14	99.6 (5)
O1W—Zn1—N1—N2	88.7 (3)	C19—N3—C13—C12	−0.7 (5)
O1^i^—Zn1—N1—N2	−103.2 (3)	C14—C12—C13—N3	−0.3 (5)
O2—Zn1—N1—N2	−1.2 (2)	C11—C12—C13—N3	173.2 (4)
O1—Zn1—N1—N2	−166.2 (3)	C13—C12—C14—C15	178.4 (5)
Zn1^i^—Zn1—N1—N2	−144.8 (2)	C11—C12—C14—C15	4.9 (7)
C8—N1—N2—C10	−175.8 (4)	C13—C12—C14—C19	1.2 (4)
Zn1—N1—N2—C10	0.3 (4)	C11—C12—C14—C19	−172.3 (4)
Zn1^i^—O1—C1—C2	29.5 (6)	C19—C14—C15—C16	1.5 (6)
Zn1—O1—C1—C2	−160.5 (3)	C12—C14—C15—C16	−175.4 (4)
Zn1^i^—O1—C1—C6	−150.3 (3)	C14—C15—C16—C17	−0.4 (7)
Zn1—O1—C1—C6	19.7 (6)	C15—C16—C17—C18	−0.7 (7)
O1—C1—C2—C3	178.2 (4)	C16—C17—C18—C19	0.5 (6)
C6—C1—C2—C3	−2.0 (7)	C13—N3—C19—C18	−175.9 (4)
C1—C2—C3—C4	−0.9 (7)	C13—N3—C19—C14	1.4 (5)
C2—C3—C4—C5	2.8 (7)	C17—C18—C19—N3	177.6 (4)
C2—C3—C4—C7	−179.0 (4)	C17—C18—C19—C14	0.7 (6)
C3—C4—C5—C6	−2.0 (7)	C15—C14—C19—N3	−179.3 (4)
C7—C4—C5—C6	179.8 (4)	C12—C14—C19—N3	−1.6 (4)
C4—C5—C6—C1	−0.8 (7)	C15—C14—C19—C18	−1.8 (6)
C4—C5—C6—C8	177.7 (4)	C12—C14—C19—C18	175.9 (4)
O1—C1—C6—C5	−177.5 (4)		

Symmetry codes: (i) −*x*+1, −*y*+1, −*z*+1.

### Hydrogen-bond geometry (Å, °)

**Table:**

*D*—H···*A*	*D*—H	H···*A*	*D*···*A*	*D*—H···*A*
O1W—H1W1···O3	0.84	1.80	2.608 (4)	161
O1W—H1W2···N2^ii^	0.84	1.87	2.703 (4)	173
N3—H3N···O4	0.88	1.95	2.822 (5)	172

Symmetry codes: (ii) −*x*, −*y*+1, −*z*+1.
